# Post-prandial glucose and insulin responses of hummus alone or combined with a carbohydrate food: a dose–response study

**DOI:** 10.1186/s12937-016-0129-1

**Published:** 2016-01-27

**Authors:** Livia S. A. Augustin, Laura Chiavaroli, Janice Campbell, Adish Ezatagha, Alexandra L. Jenkins, Amin Esfahani, Cyril W. C. Kendall

**Affiliations:** 1 Clinical Nutrition and Risk Factor Modification Center, St. Michael’s Hospital, Toronto, ON Canada; 2 Glycemic Index Laboratories Inc., Toronto, ON Canada; 3 School of Medicine, New York Medical College, Valhalla, NY USA; 4 Glycemia Consulting, 32 Ridley Gardens, Toronto, ON M6R 2T8 Canada; 5 College of Pharmacy and Nutrition, University of Saskatchewan, Saskatoon, SK Canada

**Keywords:** Chickpeas, Pulses, Blood glucose, Insulin, Glycemic index

## Abstract

**Objectives:**

Pulses are low glycemic index (GI) foods and have been associated with reduced risk of heart disease, diabetes and some cancers. However the blood glucose and insulin responses of hummus, a food containing chickpea, have not been thoroughly tested.

**Methods:**

Ten healthy subjects each consumed 11 breakfast study meals in randomized order over a period of 15 weeks. Hummus was consumed alone at three doses (2.7 g, 10.8 g and 25 g available carbohydrate [avCHO] portions) and with 50 g avCHO from white bread at three doses (2.7 g, 5.4 g and 10.8 g avCHO portions). The responses elicited by hummus alone were compared with 25 g avCHO portions of white bread, while those after hummus plus white bread were compared with 50 g avCHO from white bread. Plasma glucose and serum insulin responses were monitored over two hours and the GI and insulin index (II) calculated using standard methodology.

**Results:**

The GI and II of hummus were 15 ± 3 and 52 ± 13, respectively, and were significantly lower than white bread (*P <* 0.05). The glucose and insulin incremental area under the curve (IAUC) for hummus alone were significantly lower than white bread except for insulin IAUC of hummus 25 g avCHO. The peak rise of blood glucose and insulin after hummus were significantly lower than after white bread. Glucose and insulin IAUC after adding hummus to bread did not differ significantly from white bread alone. However the blood glucose 45 min after adding 25 g avCHO from hummus to white bread was significantly lower while at 120 min it was significantly higher than after white bread alone.

**Conclusions:**

This study demonstrated that, similar to chickpeas, hummus has a very low GI and II. Postprandial glucose responses were 4 times less than that of white bread and did not compromise insulin levels.

## Introduction

Pulses including chickpeas, split peas, lentils and beans are high in protein and dietary fiber, and have a low energy density. In epidemiological studies, pulse consumption has been associated with reduced risk of coronary heart disease (CHD) [1, 2], diabetes [3, 4] and some cancers [5]. In clinical trials, legume consumption improved glycemic control in people with diabetes [6, 7], metabolic syndrome markers in overweight and obese people [8] and satiety [9]. In early intervention studies, pulses were shown to result in lower glycemic responses [10] and to have a low glycemic index (GI) by virtue of their slow rate of carbohydrate absorption when compared to an isoglucidic standard [11]. Their low GI nature may be implicated in their protective mechanism of disease. Many studies have indeed shown that lower postprandial glycemia is associated with reduced risk of cardiovascular disease (CVD) and total mortality, independently of diabetes [12–14]. Two main mechanisms can achieve a lower GI: i) reducing the rate of carbohydrate absorption through increased content of viscous fiber, fat and/or enzyme inhibitors; ii) increasing insulin release through higher protein content. The low GI of legumes is attributed mainly to their high content of viscous fiber which delays the rate of carbohydrate absorption [15], slowly digestible starch and to their non-nutrient bioactive compounds, such as phytates, phenols, lectins and enzyme inhibitors (amylase and trypsin inhibitors), some of which may act as natural inhibitors of the digestive enzymes α-amylase and α-glucosidase [16]. The slower absorption rate makes pulses an important means of lowering the GI of the diet [7] hence the European (EASD), Canadian (CDA), and American Diabetes Associations (ADA) recommend the consumption of dietary pulses as a means of optimizing diabetes control through lowering the GI and increasing the dietary fibre content of the diet [17–19]. The American Heart Association also recommends the consumption of legumes as part of the DASH dietary approach to reduce CVD [20]. However, in North America the level of pulse consumption is low and strategies which may increase their consumption are therefore of interest. Traditional products made from chickpeas such as hummus may be a palatable and convenient way to increase legume consumption. Lentils and chickpeas are some of the lowest GI foods, however very few studies have assessed the glycemic effects of hummus. The aim of this study therefore was to determine the glycemic index of hummus and to assess its dose response effect on post-prandial blood glucose and insulin when consumed alone or when combined with a high carbohydrate, high GI food.

## Materials and methods

The study was approved by the Western Institutional Review Board® and informed consent was received from all participants.

### Study participants

A total of 10 participants were recruited, 7 men and 3 women, with mean age (± SD) of 53 ± 7 years, and mean BMI of 29.4 ± 3.8 kg/m^2^. All participants completed all test meals and no adverse events were reported. Participants were males or non-pregnant females aged 18–75 years. Participants with a known history of HIV, hepatitis, diabetes or a heart condition were excluded. In addition, those using medications or with any condition that might, either make participation dangerous to the participant or to others, or affect the results were also excluded. Participants were recruited from local advertisement and from a pool of participants who had previously indicated their willingness to participate in feeding studies.

### Dietary intervention

The study was a randomized controlled acute crossover study. The test meals were provided in two phases, with participants completing test meals 1–6 first (Phase 1) (Table 1), followed by test meals 7–11 (Phase 2) (Table 2). The order of the test meals was randomized within each phase. In addition to the test meal, each participant was provided with 250 ml of water. The aim of Phase 1 was to assess the GI, insulin index (II) and dose response of hummus fed at three levels: 1 serving (28 g, providing 2.7 g avCHO); 4 servings (112 g, providing 10.8 g avCHO); and ~9 servings (256 g, providing 25 g avCHO). In addition, each participant consumed the control white bread (providing 25 g avCHO) on three separate occasions. The aim of Phase 2 was to determine if hummus added to a high GI carbohydrate (i.e. white bread) would blunt the glycemic response. To a 50 g avCHO portion of white bread three levels of hummus was added: 1 serving (28 g); 2 servings (56 g); and 4 servings (112 g). In addition each participant consumed a control white bread (providing 50 g avCHO) on two separate occasions.

**Table 1:** Phase 1 - Dose response

Test Meal	Weight (g)	Available CHO (g)	IAUC Glucose^a^ (mmol.min/L)	IAUC Insulin^a^ (μU.min/ml)
White Bread^b^	54	25	121 ± 10a	1439 ± 432a
Hummus 28g	28	2.7	4 ± 3b	103 ± 26b
Hummus 112g	112	10.8	13 ± 4bc	641 ± 157bc
Hummus 259g	259	25	27 ± 7c	976 ± 219ac

^a^within each column, values not sharing a common superscript are significantly different (*P <* 0.05)

^b^values for White Bread are the mean of 3 tests

*IAUC* Incremental area under the curve

**Table 2:** Phase 2 - Hummus plus white bread study results

Test Meal	Weight (g)	Available CHO (g)	IAUC Glucose^a^ (mmol.min/L)	IAUC Insulin^a^ (μU.min/ml)
White Bread (WB)^b^	114	50	81 ± 20a	2618 ± 598a
WB + Hummus 28g	114	52.7	187 ± 19a	3322 ± 775a
	28			
WB + Hummus 56g	114	55.4	169 ± 21a	2365 ± 623a
	56			
WB + Hummus 112g	114	60.8	170 ± 19a	2793 ± 667a
	112			

^a^within each column, values not sharing a common superscript are significantly different (*P <* 0.05)

^b^values for White Bread are the mean of 2 tests

*IAUC* Incremental area under the curve

The hummus used in the study was Sabra Classic Hummus (Sabra Dipping Co., S. Chesterfield, VA), made primarily from chickpeas and tahini, with a macronutrient composition per serving (28 g or 2 tbsp): 3 g available carbohydrate, 1 g dietary fiber, 2 g protein, 5 g fat (SFA 0.8 g; MUFA 1.7 g; PUFA 2.6 g), and contained 50 kcal. The macronutrient profile of white bread per serving (114 g or 2 slices): 50 g available carbohydrate, 3 g fiber, 8 g protein, 2 g fat and contained 245 kcal.

### Study procedures

Eligible participants were asked to come to the clinic (Glycemic Index Laboratories, Toronto, Canada) on 11 separate occasions over a period of 15 weeks or less. Participants were asked to maintain stable dietary habits and physical activity throughout their participation in the study and to fast 10–12 h overnight prior to the morning of each test. On each test occasion, after participants were weighed, two baseline fasting blood samples were obtained by finger-prick at 5-min intervals. Finger prick serum samples were collected from hands warmed with an electric heating pad for 3–5 min prior to each sample. Blood samples were collected into 2 separate vials: one with 2–3 drops blood for glucose analysis and a second vial with 6–8 drops for insulin analysis. After the second fasting sample was collected, the participant was given the test food to eat. At the first bite a timer was started and additional samples were taken at 15, 30, 45, 60, 90 and 120 min. The participants consumed each test food within 15 min. Before and during the test, a blood glucose test record was filled out with the participant’s initials, ID number, date, body weight, test meal, beverage, time of starting to eat, time it took to eat, time and composition of last meal, and any unusual activities from the previous day. During the 2 h of the test, participants remained seated quietly. After the last blood sample had been obtained, participants were offered a snack and then allowed to leave.

### Biochemical analyses

Finger prick blood samples for glucose analysis were placed in a refrigerator and at the end of the 2-h test transferred to a −20 °C freezer until analysis (performed within 5 days from the test day). Glucose analysis was done using an YSI model 2300 STAT analyzer (Yellow Springs, OH). For insulin analysis, the microvette tubes were centrifuged and the serum transferred to labeled polypropylene tubes and stored at −20 °C. Serum insulin levels were measured using the Human Insulin EIA Kit (Alpco Diagnostics).

### Palatability

After consuming each meal, participants rated palatability using a visual analogue scale anchored by “very unpalatable” at one end (0) and “very palatable” at the other (100). Therefore, the higher the number, the higher was the perceived palatability of the product.

### Statistical analyses

Descriptive summary statistics (i.e. for continuous data number, mean, standard error of mean (SEM), were performed for all metabolites at each time-point for each test meal. The incremental areas under the plasma glucose curves (IAUC) or serum insulin curves for the food were calculated using the trapezoid rule, ignoring area beneath baseline [21]. To calculate the GI and II of the hummus, the results of the 25 g avCHO hummus and white bread test meal was used, and expressed on the glucose GI scale where glucose = 100 and white bread = 71. Results for all foods for each Phase were compared by repeated measures ANOVA for main effects of time and test meal and the time × meal interaction. The presence of a time × meal interaction means that the responses elicited by the different test meals differ significantly. If the time × mean interaction was significant, then ANOVA was conducted for each time point using the Tukey-Kramer method to adjust for multiple comparisons. Results were considered significantly different at *P <* 0.05. For the purpose of the IAUC calculation, fasting glucose or insulin was taken to be the mean of the concentrations at times −5 and 0 min. The average of the two baseline glucose measurements was used to determine the SD of the analytical variation. A second statistical analysis was performed on the percent reduction in glucose IAUC after removing values >2SD in which case excluded values were replaced by the mean of the remaining values and the error degrees of freedom in the ANOVA was reduced by the number of outliers excluded.

## Results

### Glycemic index and insulin index: phase 1

**Fig. 1:**
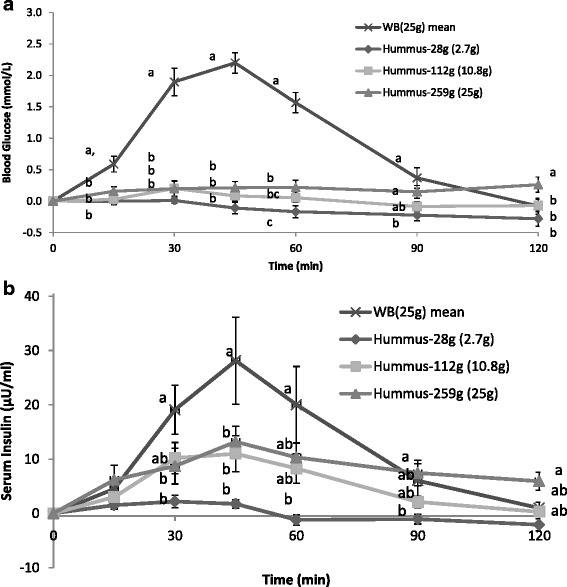
Incremental blood glucose (**a**) and insulin responses (**b**) after consumption of escalating doses of hummus (28 g, 112 g and 259 g) or white bread (WB25) containing 2.7 g, 10.8 g, and 25 g of available carbohydrate respectively. Values are expressed as Mean ± SEM. Time points not sharing a common letter are significantly different, *P <* 0.05

**Fig. 2:**
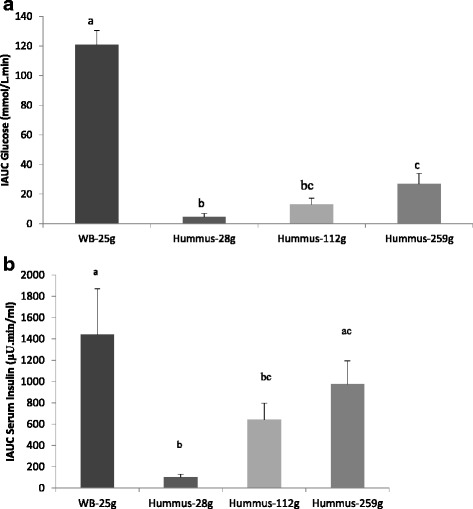
Blood glucose (**a**) and insulin (**b**) incremental area under the curve (IAUC) after consumption of escalating doses of hummus (28 g, 112 g and 259 g) or white bread (WB-25 g), containing 2.7 g, 10.8 g, 25 g and 25 g of available carbohydrate respectively. Values are expressed as Mean ± SEM. Bars not sharing a common letter are significantly different, *P <* 0.05

The blood glucose and insulin levels to the dose response hummus and white bread are presented in Fig. 1. Postprandial blood glucose values at 15, 30, 45, and 60 min after hummus consumption at any dose were all significantly lower than with white bread (Fig. 1a). Postprandial peak serum insulin levels for all hummus meal at 45 min were significantly lower than that for white bread (Fig. 1b). Glucose IAUC of white bread was significantly higher than the IAUC of any of the hummus meals. In addition, the IAUC of the 28g hummus meal was significantly lower than that of the 259 g hummus meal (*P <* 0.05), (Fig. 2a). The insulin IAUC of white bread was significantly higher than hummus 28 g and 112 g but not significantly different than hummus 259 g which had the same amount of available carbohydrates as the white bread (Fig. 2b). There was also a significant and strong correlation between amount of available carbohydrate in the hummus test meals and the blood glucose IAUC (r = 0.995, *P <* 0.001).

The GI and II of hummus were calculated from data of the 259 g (25 g avCHO) serving test meals (Fig. 1). The GI of hummus was found to be 15 ± 3 and significantly lower than white bread (*P <* 0.05), and hence hummus falls in the low GI category, i.e. GI ≤ 55 [22]. The mean II of hummus was 52 ± 13 and significantly lower than white bread (*P <* 0.05).

There were no statistically significant differences in palatability scores between the meals (data not shown).

### Hummus plus white bread - phase 2

**Fig. 3:**
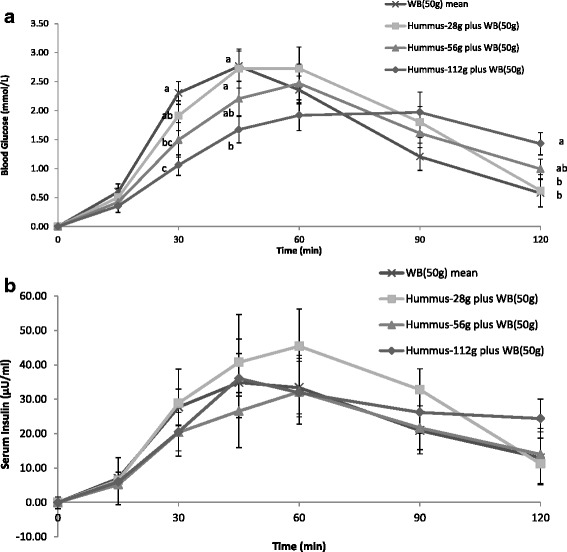
Incremental blood glucose (**a**) and insulin (**b**) responses after consumption of escalating doses of hummus (28 g, 56 g and 112 g) containing 2.7 g, 5.4 g and 10.8 g of available carbohydrate, respectively, plus white bread or white bread alone (WB50) containing 50 g of available carbohydrate. Values are expressed as Mean ± SEM. Bars not sharing a common letter are significantly different, *P <* 0.05

The blood glucose and insulin response curves for the 1, 2 and 4 servings of hummus consumed with a 50 g avCHO portion of white bread are presented in Fig. 3. The postprandial blood glucose levels were significantly lower than the white bread control at 30 min and 45 min after consumption of 112 g hummus + WB meal and at 30 min after consumption of 56 g hummus + WB (*P <* 0.05) (Fig. 3a). At 120 min the only significant difference was between 112 g hummus + WB and white bread alone, where the highest serving of hummus maintained blood glucose at a higher level (*P <* 0.05) (Fig. 3a). There were no significant differences however between the glucose IAUCs (Table 2). Postprandial serum insulin levels were not significantly different between meals at any time point nor were the insulin IAUCs (Fig. 3b and Table 2). There were no statistically significant differences in palatability scores between the meals (data not shown).

## Discussion

The glycemic response of hummus consumed alone was less than one quarter that of white bread for the same amount of available carbohydrate. The GI of hummus was therefore very low (15 GI points on a glucose scale or 22 on a white bread scale) and was achieved without a significant increase in insulin levels and with lower and sustained blood glucose excursions. This suggests a slow release mechanism where the absorption rate of the carbohydrate component is slowed and prolonged over time possibly owing to the high viscous fiber content [15], the high ratio of amylose starch to amylopectin [23], and possibly the presence of enzymes inhibitors [24]. The alpha-glucosidase inhibitor acarbose has been shown to decrease incident diabetes in people with impaired glucose tolerance individuals and decrease cardiovascular events [25, 26]. The GI of hummus was less than half of that of chickpeas alone (15 versus 36) [22]. The high and healthy (MUFA and PUFA) fat content of the hummus, 5 g/serving, is 6 times higher than that of chickpeas alone and may partly account for the very low GI observed in this study. Dietary fat delays gastric emptying thereby slowing carbohydrate absorption [27, 28]. A study of low GI/high unsaturated fat diets demonstrated several health advantages of this dietary combination, including improvements in glycemic control and CVD risk score, particularly in those with metabolic syndrome components (e.g. central obesity) [29]. The GI of hummus in our study (GI = 15) was similar to another hummus dip reported in the International Tables of GI as item n.1097 [22]. Other investigators found a slightly lower GI for hummus (GI = 6) [30]. Food processing may alter the GI of a food. A 35 % higher GI was seen with canned chickpeas than with chickpeas cooked from dry [22, 31] and as much as 3-fold higher compared to chickpeas cooked for a short time (35 min) [22, 32], albeit in this study the blood measurements were taken for one hour less than the standard two hours required for healthy people by the standard GI protocol [33]. Cooking increases hydration and gelatinization of the starch molecule making it more readily available for enzyme digestion and hence absorption of glucose. Cooking may also reduce the content of phytates and lectins which are inversely related to the glycemic response [24]. It is interesting to note that in our study the highest servings of hummus, either alone or with white bread, not only induced lower blood glucose excursions compared to white bread alone but these responses were obtained with an insulin-sparing effect (at 45 min) and were sustained over time which means that after two hours from meal consumption blood glucose was maintained slightly above baseline. This may have several beneficial metabolic and health implications: suppression of plasma free fatty acids, glucagon and growth hormone, beneficial second meal effects [34, 35], decreased hunger and possible effects on cognitive functions [36]. Furthermore, hypoglycemia is related to higher cardiovascular complications in people with type 2 diabetes [37] hence hummus consumption may help to avoid hypoglycemia. Future studies should assess the effect of hummus on incretin levels and markers of satiety acutely in individuals with diabetes and those without. Longer-term clinical studies should also be undertaken to assess the effect of hummus on body weight and markers of cardiometabolic health.

## Conclusion

This study demonstrated that, similar to chickpeas, hummus has a very low GI and II. Postprandial glucose responses were 4 times less than that of white bread and did not compromise insulin levels. Lower glycemic and insulinemic responses are related to better health outcomes and low GI diets have been inversely associated with type 2 diabetes, CVD and some cancers [38–40], with improved glycemic control [7, 41, 42], body fat and weight management [43–46]. Diabetes and heart association guidelines promote the consumption of pulses and unsaturated fat as well as low GI food options [18, 20, 47]. Hummus has the advantage of having a complete and healthy macronutrient profile and hence it can be consumed as a meal as well as a snack or a side dish replacing high GI starches (e.g. rice or potatoes). Hummus is a healthy food that fits with dietary guidelines aimed at reducing risk of CVD and diabetes complications.
